# Prevalence and risk factors of pre-senile lens opacities in the 1969-73 Vellore Birth Cohort

**DOI:** 10.1038/s41433-025-03836-9

**Published:** 2025-06-13

**Authors:** Padma Paul, Belavendra Antonisamy, Neena John, Andrew Braganza, Thomas Kuriakose, Rita Isaac, Lekha Abraham, Anika Amritanand, Prasanna Samuel, Hepsy Y. Chelliah, Mahasampath Gowri S., Nancy Magdalene, Jophy Philips Cherry, Thomas V. Paul, Felix Jebasingh, Geetanjali Arulappan, Nihal Thomas, Senthil K. Vasan, G. V. S. Murthy, Clare Gilbert

**Affiliations:** 1 Department of Ophthalmology, Christian Medical College and Hospital, Vellore, India; 2 Department of Biostatistics, Christian Medical College and Hospital, Vellore, India; 3 Indian Institute of Public Health, Hyderabad, India; 4 RUHSA Department, Christian Medical College and Hospital, Vellore, India; 5 Department of Endocrinology, Christian Medical College and Hospital, Vellore, India; 6 Department of Clinical Biochemistry, Christian Medical College and Hospital, Vellore, India; 7 Centre for stem Cell Research, CMC Vellore (An Unit of inStem Bengaluru, CMC Vellore, VELLORE, India; 8 Salford Royal NHS foundation Trust, Manchester, United Kingdom; 9 International Centre for Eye Health, Department of Clinical Research, London School of Hygiene & Tropical Medicine (LSHTM), London, UK

**Keywords:** Diseases, Lens diseases

## Abstract

**Purpose:**

To estimate the prevalence and determine predictors of lens opacities (LO) among South Asian Indians aged 41–44 years.

**Methods:**

This cross-sectional study included 1080 participants from the Vellore Birth Cohort, Vellore, South India. All underwent anthropometric measurements, detailed ophthalmic examination including assessment of LO by LOCS III classification and biochemical metabolic measurements. ‘Any cataract’ was defined as any opacity type with a score of >2 or evidence of cataract surgery in either eye. Data collected included information on ocular history, life-style factors, socio-economic and educational status, cooking fuel and sunlight exposure. Multivariable logistic regression analysis was used to examine the association between risk predictors and LO.

**Results:**

The mean age (SD) of participants was 41.8 (1.0) years; 53.8% were male and 50% were rural residents. The overall prevalence of ‘any cataract’ was 13.8% (148/1075, 95% confidence interval (CI) 11.8,16.0). The types of cataract were nuclear 59.1%, cortical 16.9%, posterior subcapsular 4.1%, mixed cataracts 18.9% and pseudophakia 0.7%. Increased risk for LO was observed with a history of asthma (OR 4.51; 95% CI 2.1, 9.7), HbA1C of ≥6.5% (OR 2.29; 95% CI 1.4, 3.7), hypertension (OR 1.73; 95% CI 1.1, 2.7) and, in a subgroup (*n *= 372), lower 25(OH) vitamin D levels (≤20 ng/dL)(OR 5.56; 95% CI 2.3, 13.2).

**Conclusion:**

The high prevalence of LO at a relatively young age in South Asian Indians suggests earlier onset of ageing. History of asthma, higher HbA1C, hypertension and lower 25(OH) vitamin D levels were associated with LO.

## Introduction

The global increase in population and life expectancy have led to an increase in the number of people who are blind, from 36 to 43.3 million, despite a reduction in the global prevalence [1, 2]. Age related cataract (lens opacity, LO), accounts for almost 40% of all blindness and 28% of all moderate and severe visual impairment (VI) among those aged 50 years and above, with higher proportions in Asia and lower proportions in Africa [2, 3]. Recognized risk factors for LOs include advancing age, specific ethnic groups such as South Asians [4, 5] and modifiable risk factors such as low socio-economic status, smoking, ultraviolet light exposure, obesity, asthma, hypertension, underlying metabolic disorders (diabetes) and exposure to drugs such as steroids [6]. In Asian Indians, the onset of LOs is earlier than in high income countries [7, 8], which may reflect earlier onset of ageing [9] due to greater exposure to risk factors [10, 11]. Lens opacities have a multifactorial aetiology, with genetic and environmental factors interacting to increase oxidative stress in the lens. Higher levels of hydrogen peroxide, superoxide (O_2_^-^) and hydroxyl (OH) free radicals in the lens and aqueous humour are associated with LO [12]. Cells in the lens proliferate throughout life and LOs reflect a lifetime of insults, including oxidative stress [5]. However, randomized clinical trials of antioxidant vitamin supplements (i.e., A, C and E) have not shown any beneficial effects on the incidence or progression of LOs [13] including one from India [14]. There is less evidence of whether vitamin-D deficiency/insufficiency is associated with LOs.

Birth cohort studies have the potential to provide unique insights as exposure to risk factors can be explored across the life course. In this paper we report the prevalence of LO and exposure to risk factors in adults who were recruited to the Vellore Birth Cohort 41–45 years earlier.

## Methods

Participants for the current study were traceable members of a subset of the Vellore Birth Cohort (VBC) in which all infants born to women in defined areas of Vellore town and three adjoining rural villages in Tamil Nadu, India, between 1969 and 1973 were included. Subsequently 10,670 singleton live births were followed up during infancy, adolescence and adulthood [15].

### Study population

This was a cross-sectional study of adults aged 41–44 years from the Phase-6 follow-up (2013-14) of VBC recruited from one urban and three rural areas nearby. The cohort is described in detail elsewhere [16]. The study adhered to the guidelines of the declaration of Helsinki and was approved by the ethics committees of Christian Medical College, Vellore (IRB min no.7765 dt 22/2/2012), and Public Health Foundation of India.

### Study participants

**Fig. 1:**
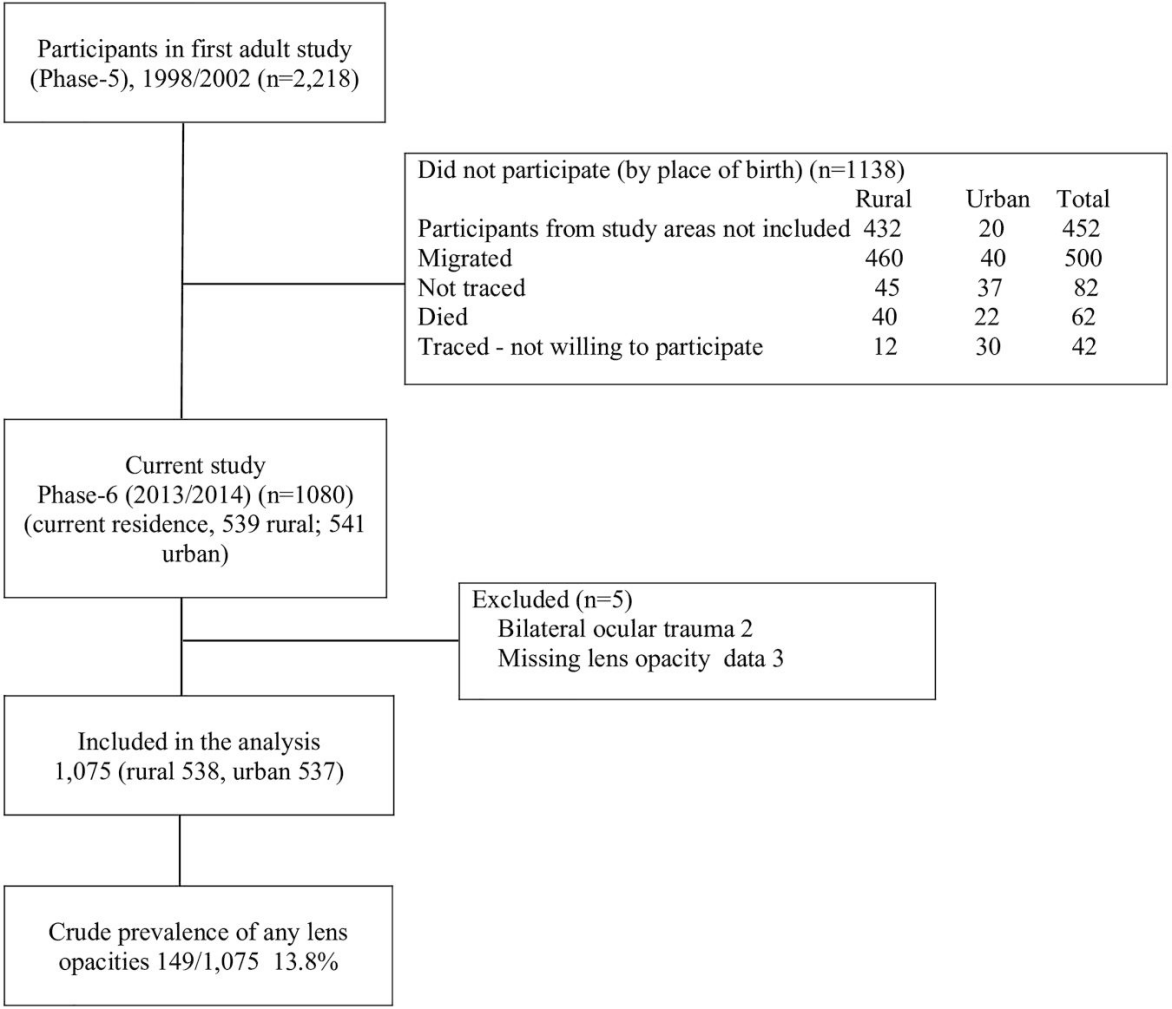
Flow of the study.

One thousand and eighty of the 2218 traced cohort members who took part in the Phase 5 of the VBC study (1998–2002) were included (Fig. 1) [16].

After obtaining written informed consent trained health workers collected data on socio- demographic status, life-style characteristics (smoking, alcohol consumption) and daily hours of sunlight exposure in participants’ homes using questionnaires. Socioeconomic status, educational status and the physical activity score were determined as described earlier [16–19]. Smoking status was defined as current smokers of cigarettes or ‘beedis’. Current alcohol consumption was defined as consumption of any local or imported spirits, beer or wine. The main type of fuel used for cooking in their household was noted.

### Ophthalmic history and examination

Participants attended the Department of Ophthalmology, Christian Medical College (CMC), Vellore for examination. Following a brief ocular history, distance visual acuity (VA) (uncorrected, presenting and best corrected after retinoscopy and subjective refraction), was measured using a self-illuminated logMAR visual acuity chart at 4 meters, and near vision was tested using a logMAR chart for near vision at 40 cm. Presenting VA in the better eye for distance was categorized according to World Health Organization (WHO) as ’good’ (≥6/12), ‘mild visual impairment’ (VI) (<6/12–6/18), ‘moderate VI’ (<6/18–6/60), ‘severe VI (<6/60–3/60) or blind (<3/60) after conversion to Snellen equivalents. Good near vision was defined as a corrected acuity of N8 equivalent (logMAR 1.0 M) or more in the better eye. A comprehensive ophthalmic examination was performed by a trained ophthalmologist using a Haag Streit slit lamp which included: the intraocular pressure (IOP) measurement using Goldman Applanation tonometry; dilated fundus examination using a 90 D (Volk) lens, and grading LOs using a Lens Opacities Classification System III (LOCS) standard plate [20]. Axial length was measured using ultrasound biometry (Ocuscan, ALCON).

‘Any cataract’ was defined as significant LO or evidence of cataract surgery as an adult in one or both eyes. Using LOCS III classification significant LO was defined as a score of >2 for each type of opacity i.e., for nuclear opalescence (NO), nuclear colour (NC), cortical opacity (CO) or posterior sub-capsular opacity (PSCO); NO or NC of >2 was reported as NC. Those with more than one type were classified as ‘mixed LO’. Inter-observer agreements for 60 participants were : kappa 0.93 (95% CI 0.8–1.0) for right eyes and 0.87 (95% CI 0.68–1.0) for left eyes. If a participant was pseudophakic/aphakic in one eye, the LOCS III grading in the other eye was used. Pseudophakia/aphakia was used if both eyes had undergone cataract surgery, or if the unoperated eye had a LO score of ≤2. If one eye had a condition precluding assessment of LOCS III or evidence of unilateral injury then scores from the other eye were used.

### Clinical parameters and biochemical evaluation

Anthropometry included measurements of height, weight, waist circumference (WC), hip circumference (HC) and blood pressure (BP) using standard protocols. Body mass index (BMI) was calculated as the ratio of weight (kg) to height^2^ (m^2^). We used WHO definitions for underweight, normal, overweight and obesity [16]. The average of three measurements was used in the analysis. Hypertension was defined as systolic blood pressure (SBP) ≥ 140 mmHg and diastolic blood pressure (DBP) ≥ 90 mmHg and/or being on medication for hypertension [21]. Blood samples were assayed for fasting plasma glucose by hexokinase method, lipids by colorimetry using Roche Chemistry analysers and glycosylated haemoglobin (HbA1C) by HPLC using Biorad Variant II. As many people with diabetes in India are not diagnosed diabetes was defined as HB A1C of ≥6.5% [22]. Serum 25(OH) vitamin-D levels were measured by electrochemiluminescence assay using Cobas e170 in a subset of 372 participants on whom values were available as they were taking part in another study at the same time [23]. Serum levels ≤20 ng/dL were categorized as deficient [24]. Participants with undiagnosed diabetes or with ocular morbidity requiring treatment were referred to respective clinics at the hospital. 

### Statistical analysis

Participants’ characteristics are presented as means with standard deviation (SD) for normally distributed variables; median (inter-quartile range; IQR) for skewed variables, and proportions for categorical variables. Baseline characteristics are summarized using two sample t-tests and chi-square tests stratified by gender and place of residence. Risk factors for LO were chosen based on clinical importance. Univariate and multivariable logistic regression analyses were used to study predictors of LO and the results are presented as odds ratios (OR) and 95% confidence intervals (CI). All variables were entered simultaneously in the multivariate model which included age, gender, education and current smoking, alcohol consumption, household possession score, hours outdoor, cooking fuel used, history of asthma, HbA1c, hypertension, body mass index, axial length and physical activity score. A subgroup analysis (*n *= 374) was undertaken to explore the association between serum 25(OH) vitamin D levels and LO in individuals on whom 25(OH) vitamin D was measured [23]. All statistical analyses were performed using Stata/IC version 16 (StataCorp. 2019. College Station, TX: LLC).

## Results

A total of 1080 traced cohort members who agreed to participate were examined; five were excluded (two had history of bilateral eye injuries and three had missing LOCS III data) leaving 1075 for analysis (Fig. 1). The mean age (SD) of participants at examination was 41.8 (1.0) (range 41–44) years and 53.8% (*n *= 578) were male (Table 1). The mean BMI (SD) was 25.4 (4.8) kg/m^2^ (in the overweight range). Only men reported smoking (32.5%) and consuming alcohol (45%).

**Table 1:** Characteristics of the study population and risk factors for lens opacities, by sex and place of residence.

Variables	Male (*n *= 578)		Female (*n *= 497)		Male vs female	Rural vs urban
	Rural (*n *= 289)	Urban (*n *= 289)	Rural (*n *= 249)	Urban (*n *= 248)	*p* value	*p* value
Age (mean, SD) years	41.9 (0.9)	41.5 (1.0)	42.0 (0.9)	41.7 (1.0)	0.182	<0.001
Educational status (*N*, %)						
Up to middle school	136 (47.1)	122 (42.2)	173 (69.5)	125 (50.4)	<0.001	<0.001
High school and above	153 (52.9)	167 (57.8)	76 (30.5)	122 (49.6)		
Household asset score (SES)						
1 (lowest)	109 (37.7)	42 (14.5)	112 (45.0)	40 (16.1)	0.228	<0.001
2	72 (24.9)	65 (22.5)	51 (20.5)	47 (19.0)		
3	65 (22.5)	80 (27.7)	57 (22.9)	74 (29.8)		
4 (highest)	43 (14.9)	102 (35.3)	29 (11.7)	87 (35.1)		
Body mass index(kg/m^2^) (*N*, %)^a^						
<18.5	24 (8.3)	18 (6.3)	18 (7.2)	11 (4.4)	<0.001	<0.001
18.5–24.9	155 (53.6)	124 (43.1)	104 (41.8)	63 (25.4)		
25.0–29.9	94 (32.5)	108 (37.5)	85 (34.1)	96 (38.7)		
≥30.0	16 (5.5)	38 (13.2)	42 (16.9)	78 (31.5)		
Physical activity score (median, IQR)	1290 (825, 1498)	1015 (780, 1410)	1758 (1438, 2114)	1640 (1333,2042)	<0.001	0.014
Smoking status (*N*, %) Yes	70 (24.2)	117 (40.5)	0 (0.0)	0 (0.0)	<0.001	<0.001
Alcohol status (*N*, %) Yes	151 (52.3)	109 (37.7)	0 (0.0)	0 (0.0)	<0.001	0.003
Hours outside home (*N*, %)						
1–4 h	109 (37.7)	35 (12.1)	160 (64.3)	58 (23.4)	<0.001	<0.001
5–9 h	128 (44.3)	52 (17.9)	61 (24.5)	8 (3.2)		
10 and above	52 (18.0)	202 (70.0)	28 (11.2)	182 (73.4)		
Cooking fuel (*N*, %)						
Liquefied petroleum gas	206 (71.3)	255 (88.2)	184 (73.9)	214 (86.3)	0.826	0.826
Wood	78 (27.0)	18 (6.2)	62 (24.9)	16 (6.5)		
Others (kerosene, biogas)	5 (1.7)	16 (5.6)	3 (1.2)	18 (7.3)		
Hip circumference (mean, SD) cm	89.2 (7.2)	92.7 (8.6)	92.7 (10.3)	99.3 (11.1)	<0.001	<0.001
Waist circumference (mean, SD) cm	87.8 (11.1)	92.2 (12.1)	81.7 (12.0)	87.5 (11.6)	<0.001	<0.001
Waist hip ratio	0.98 (0.06)	0.99 (0.06)	0.88 (0.08)	0.88 (0.07)	<0.001	0.25
HbA1C (*N*, %)						
<6.5%	246 (85.1)	248 (85.8)	229 (92.0)	217 (87.9)	0.028	0.446
≥6.5%	43 (14.9)	41 (14.2)	20 (8.0)	30 (12.1)		
Hypertension (*N*, %)						
Yes	77 (26.6)	84 (29.1)	33 (13.3)	39 (15.7)	<0.001	0.328
Intraocular pressure (mean, SD)						
Right eye Left eye	13.4 (3.0)	13.9(3.0)	13.0(2.9)	13.9(2.8)	0.346	0.0002
	13.6 (3.1)	14.2(3.0)	13.5(3.0)	14.1(2.8)	0.630	0.004
Lens opacity type (*N*, %)						
No opacity	239 (82.7)	252 (87.2)	224 (90.0)	213 (85.9)	0.522	0.709
Nuclear	28 (9.7)	22 (7.6)	14 (5.6)	24 (9.7)		
Cortical	11 (3.8)	6 (2.1)	5 (2.0)	3 (1.2)		
Posterior	2 (0.7)	1 (0.4)	1 (0.4)	2 (0.8)		
Mixed	9 (3.1)	8 (2.8)	5 (2.8)	6 (2.4)		
Any lens opacity (*N*, %)	50 (17.3)	37 (12.8)	26 (10.4)	35 (14.1)	0.187	0.732

*IQR* interquartile range.

^a^Data missing for one participant.

Women had higher physical activity scores than men and urban and rural women spent longer outside the home than their male counterparts. Most participants (80%) used liquid petroleum gas (LPG) as a cooking fuel. Approximately 3% had a history of asthma, 12.5% had an HbA1c of ≥6.5% and 21.7% had hypertension.

### General ocular findings

Family history of spectacle use / holding things close to see was reported in one or both parents by 26.1%, in sibling alone in 4.5%, children alone in 2.5% and a combination of more than one first degree relative in 16.8% of participants. Reported spectacle use for near alone, distance alone and for both was 7.7%, 5.5% and 2.2% respectively. Self-reported ocular history included trauma in one eye [33, 3.1%], night blindness [6, 0.6% (mostly women)], surgery in either eye for cataract, glaucoma, or trauma [8, 0.7%] and current use of eye drops [8, 0.7%].

Among the 1065 participants with VA data, 95% had ‘good’ VA, 2% had ‘mild VI’ and 3% had ‘moderate VI’ ; 99.7% had normal near vision with correction. The mean (SD) IOP was 13.7 (2.8) mm Hg.

### Lens opacities

The overall prevalence of ‘any cataract’ was 13.8% (95% CI 11.8, 16.0) [men: 15.1% (95% CI 12.2,18.2); women: 12.3% (95% CI 9.5,15.5)]. Combining gender and place of residence the prevalence was as follows: rural men, 17.3% (50/289), rural women 10.4% (26/250), urban men 13.2% (38/288) and urban women 14.2% (35/247). There was no significant difference by sex (*P* = 0.16) or place of residence (*P* = 0.83). Nuclear cataract was the commonest type of LO (59.1%) followed by cortical (16.9%), posterior subcapsular (4.1) and mixed opacities (18.9%). Only 0.7% were pseudophakic in both eyes.

In unadjusted logistic regression analysis, higher household asset scores and higher educational status were significantly associated with LO, but were not significant in the multivariable model (Table 2). The following remained statistically significant in the multivariable model: higher HbA1C (OR 2.29; 95% CI 1.4, 3.7), hypertension (OR 1.73; 95% CI 1.1, 2.7) and a history of asthma (OR 4.51; 95% CI 2.1, 9.7). Positive history of rheumatoid arthritis (*P* = 0.31) and use of systemic glucocorticoids (*P* = 0.92) were not associated with LO.

**Table 2:** Univariate and multivariable analysis of risk factors for lens opacity.

Risk factors	Lens opacity (LOCS III) (*n *= 1075)		Unadjusted		Adjusted^a^	
	Absent (*n *= 927) *n* (%)	Present (*n *= 148) *n* (%)	OR (95% CI)	*p*- value	OR (95% CI)	*p*- value
Age (mean, SD) years	41.8 (1.0)	41.8 (0.9)	1.09 (0.9, 1.3)	0.36	1.10 (0.9, 1.3)	0.32
Sex						
Male	491 (84.9)	87 (15.1)	1.27 (0.9, 1.8)	0.19	1.17 (0.7, 1.9)	0.54
Female	433 (87.7)	61 (12.3)	1.00		1.00	
Education (*N*, %)						
Up to middle school	494 (88.9)	62 (11.2)	1.00		1.00	
High school and above	433 (83.4)	86 (16.6)	1.58 (1.1, 2.2)	0.01	1.34 (0.9, 1.9)	0.16
Household assets (SES) (*N*, %)						
1 (lowest)	266 (87.8)	37 (2.2)	1.00	0.02^c^	1.00	
2	209 (88.9)	26 (11.1)	0.89 (0.5, 1.5)		0.71 (0.4, 1.3)	0.28
3	241 (87.3)	35 (12.7)	1.04 (0.6, 1.7)		0.63 (0.4, 1.2)	0.16
4 (highest)	211 (80.8)	50 (19.2)	1.70 (1.1, 2.7)		1.01 (0.5, 1.9)	0.98
Body mass index (kg/m^2^) (*N*, %)^b^						
<18.5	63 (88.7)	8 (11.3)	0.74 (0.3, 1.6)		0.94 (0.4, 2.1)	0.88
18.5-24.9	381 (85.4)	65 (14.6)	1.00	0.67^c^	1.00	
25.0–29.9	336 (87.7)	47 (12.3)	0.82 (0.5, 1.2)		0.64 (0.4, 0.9)	0.05
≥30.0	146 (83.9)	28 (16.1)	1.12 (0.7, 1.8)		0.84 (0.5, 1.4)	0.54
Physical activity score, median	1425	1320	0.99 (0.9, 1.0)	0.098	0.99 (0.9, 1.0)	0.69
(IQR)	(1011, 835)	(910, 1746)				
Current smoking (*N*, %)						
No	764 (86.0)	124 (14.0)	1.00		1.00	
Yes	163 (87.2)	24 (12.8)	0.91 (0.6, 1.4)	0.68	0.75 (0.4, 1.3)	0.31
Alcohol consumption (*N*, %)						
No	705 (86.5)	110 (13.5)	1.00		1.00	
Yes	222 (85.4)	38 (14.6)	1.10 (0.7, 1.6)	0.650	0.91 (0.5, 1.5)	0.72
Hours outdoors (*N*, %)						
1–4	308 (85.1))	54 (14.9)	1.00	0.84^c^	1.00	
5–9	225 (90.4)	24 (9.6)	0.61 (0.4, 1.0)		0.56 (0.3, 0.9)	0.05
10 and above	394 (84.9)	70 (15.1)	1.01 (0.7, 1.5)		0.99 (0.6, 1.5)	0.95
Cooking fuel used (*N*, %)						
Liquefied petroleum gas	736 (85.7)	123 (14.3)	2.17 (0.7, 7.1)	0.20	1.75 (0.5, 6.0)	0.38
Wood	152 (87.4)	22 (12.6)	1.88 (0.5, 6.6)	0.32	1.99 (0.5, 7.4)	0.30
Others	39 (92.9)	3 (7.1)	1.00		1.00	
History of asthma (*N*, %)						
Yes	20 (60.6)	13 (39.4)	4.36 (2.1, 8.9)	<0.001	4.51 (2.1, 9.7)	<0.001
No	906 (87.0)	135 (13.0)	1.00		1.00	
HbA1c (*N*, %)						
<6.5%	828 (88.1)	112 (11.9)	1.00		1.00	
≥6.5%	98 (73.1)	36 (26.9)	2.72 (1.8, 4.2)	<0.001	2.29 (1.4, 3.7)	0.001
Hypertension (*N*, %)						
Yes	184 (79.0)	49 (21.0)	2.00 (1.4, 2.9)	<0.001	1.73 (1.1, 2.7)	0.015
No (ref)	743 (88.2)	99 (11.8)	1.00		1.00	
Axial length (mm), mean (SD)	22.9 (0.9)	23.0 (1.0)	1.16 (0.9, 1.4)	0.11	1.17 (0.9, 1.4)	0.13

^a^Adjusted for age, sex, BMI, education, household possession score, current smoking, alcohol consumption, hours outdoor, cooking fuel used, HbA1c, hypertension and axial length.

^b^Data missing for one participant.

^c^*p*-value for trend test.

In the subgroup analysis of 372 individuals, LOs were significantly associated with low 25(OH) vitamin D levels (OR 5.56; 95% CI 2.3, 13.2) (*P* < 0.001) (Table 3). The proportion of people in the population with LO from exposure to vitamin D deficiency which could be prevented by correcting vitamin D deficiency was 56% (i.e., the population attributable risk) (Supplementary Table 1). A sensitivity analysis comparing the sub-group with vitamin D data (*n *= 372) with those without (*n *= 703) showed no significant differences in most characteristics except education and type of LO. Those without vitamin D levels were better educated (high school and above; 48.4% versus 48.1%, *P* = 0.01) and had a higher prevalence of any LO (15.5% versus 10.5%, *P* = 0.02).

**Table 3:** Vitamin D deficiency (≤20 ng/dL) and presence of lens opacity (LOCS III) (sub-group analysis, *n *= 372).

Vitamin D (ng/ml) (*N*, %)		Lens opacity (LOCS III)		Unadjusted		Adjusted^a^	
	Total	No	Yes	OR (95% CI)	*p*-value	OR (95% CI)	*p*-value
≤20 ng/dL	155 (41.7)	126 (81.3)	29 (18.7)	4.76 (2.2,10.1)	<0.001	5.56 (2.3,13.2)	<0.001
>20 ng/dL (ref)	217 (58.3)	207 (95.4)	10 (4.6)	1.0		1.0	

^a^Adjusted for age, sex, BMI, education, household possession score, current smoking, alcohol consumption, hours outdoor, cooking fuel used, HbA1c, hypertension and axial length.

To assess the representativeness of participants in our study from cohort members examined in 1998–2002 (*n *= 2218) [19] the age, gender, place of residence, educational status and SES among those examined in the current study (2013/2014) were compared with those who were not. There were significant differences only in place of birth (rural/urban; (*P* < 0.001) and SES (*P* < 0.001) and not in age, sex and educational status (Supplementary Table 2).

## Discussion

Lens opacities increase over the age of 50 years and only a few studies have reported the prevalence of LO among individuals aged less than 50 years (Table 4) [25–36]. Our prevalence estimate (13.8%) is comparable to the south Indian Aravind Comprehensive Eye Study (AECS), which used similar methods (age range 40–49 years, 15.7%) [35], but was higher than in a Chinese study of 45–49 year olds (5.9%, 95% CI 4.9–7.0) [29]. The prevalence of LOs using the LOCS II grading of 2 or more in the Barbados Eye studies (age range 40–49 years) were between 3.0 and 4.7% [25, 28, 37]. Comparing prevalence estimates between studies needs caution, due to methodological differences in the definitions and classification systems used for LOs (Table 4). However, the prevalence does seem to be lower in high-income countries than in middle-income countries, which may be explained by lower exposure to modifiable risk factors, such as lifestyle factors, and better control of blood glucose amongst people with diabetes.

**Table 4:** Prevalence of lens opacities in population-based studies reporting a similar age group.

WHO Region	Country	Year	Age group (years)	Sample size	Rural/urban residence	LO grading system ^a^	Prevalence (%)(95% CI)	Commonest type of LO
The Americas	USA(25)	2003	40-49	2363	Urban	LOCS II (2/2/2)	3 (2.3-3.7)	CO (1.7%)
European	Italy (26)	1995	40-49	278	Urban	LOCS II (2/2/2)	4 (2.8–5.1)	N (1.5%)
African	Tanzania (27)	2001	40-49	1339	Rural	WHO simplified grading system	4.5 (3.4-5.6)	CO (2.4%)
The Americas	Barbados (28)	1996	40-49	1333	^b^	LOCS II (2/2/2)	4.7	CO (3.9%)
Western Pacific	China (29)	2012	45-49	1917	Rural	LOCSIII (2/2/2)	5.9 (4.9-7)	CO (3.5%)
South-East Asian	Myanmar (30)	2007	40-49	657	Rural	LOCS III (4/2/2)	6.1 (4.3-7)	NO (2.4%)
Western Pacific	Singaporean Malay(31)	2004–06	40-49	813	Urban	WISCONSIN	6.2 (4.5-7.9)	CO (4.2%)
Western Pacific	Singaporean Chinese(32)	1997-8	40-49	270	^b^	LOCS III (4/2/2)	7.0	CO (3.0%)
South-East Asian	Sri Lanka (33)	2006-7	40-49	331	Rural	LOCS III (4/2/2)	9.4 (6.3-12.5)	^c^
Eastern Mediterranean	Pakistan (34)	2007	40-49	3567	Urban /Rural	Mehra Minassian	10.6 (9.6-11.7)	^c^
**South-East Asian**	**India, Vellore**	**2014**	**41-45**	**1075**	**Rural/urban**	**LOCSIII (>2/2/2)**	**13.8 (11.8–16.0)**	**N (8.2%)**
South-East Asian	India, Madurai (35)	1995-7	40-49	2061	Rural	LOCS III (3/3/2)	15.7 (14.1-17.3)	NO (8.2%)
South-East Asian	Indonesia (36)	2003	40-49	198	Rural	LOCS III (4/4/2)	24.3 (0-100)	Mixed 16%

*LO* lens opacity, *CO* cortical, *NO* nuclear opalescence, *N* nuclear.

^a^Grading system cutoffs for nuclear/cortical/posterior sub capsular cataracts.

^b^Not mentioned.

^c^Not available for this age group.

In our study, nuclear LOs were the commonest type (8.1%), which is similar to other Indian studies such as AECS (8.2%) [35], but higher than in the Andhra Pradesh Eye Disease Survey (APEDS)(3.5%) [38]. In high-income countries cortical LO are commoner in both younger and older populations [28]. Different LO types may be associated with specific risk factors, the most commonly reported being cortical LO and high UVB exposure [39]. However, in our study the number of participants with LOs were too few for analysis by type of LO.

Men had a slightly higher prevalence of LO than women, but this was not statistically significant. This differs from other studies where women generally have a higher prevalence, particularly earlier studies [28, 40, 41]. Reasons for the gender difference are not fully understood, but may be due to a fall in oestrogen-mediated anti-ageing effects on the lens in women [42]. Less pronounced gender differences in LO in younger populations were also reported from the Swedish national cataract register [43].

The Beaver Dam Eye Study showed a U-shaped relationship between SES and cataracts, with higher frequencies at extremes of SES [44], reflecting different exposure to risk factors amongst those very poor and very affluent. Our study did not show any significant relationship with SES, despite a detailed SES assessment using multiple indicators such as household assets and education. This is in contrast to APEDS, where the prevalence of LOs was higher among those with a lower SES based on monthly income [38]. The different indicators used to calculate SES may explain the differences.

There is clear evidence from observational studies that smoking increases the risk of cataract [45], which is mediated by oxidative damage from smoke constituents [46]. In our study there was no association with between LO and smoking as has been reported from East Asia [40, 47] but other studies in India have shown an association [38, 41]. There was also no association between biomass cooking fuels and LO, unlike other studies from India [48]. Possible explanations for both of these findings are the relatively young age of our study participants who would have had fewer packs-years of smoking exposure and fewer cumulative years of exposure to biomass cooking fuels due to the use of gas for cooking.

The increased risk of LO among individuals with asthma has been reported previously [49, 50], which may reflect steroid use. In a large general practice study in the United Kingdom, (*n *= 201,816; age 3–90 years), corticosteroid use was associated with increased cataract risk (relative risk 1.3) but this was not evident in those under the age of 40 years [51]. Our study lacked information on duration of steroid use.

Previous studies show variable associations between obesity and LOs [40, 52, 53]. Pooled estimates from a meta-analysis of 17 studies, including one from Asia, demonstrated a 2% increase in age-related cataracts with every 1 kg/m^2^ increase in BMI for PSC only, but the pooled effect showed a weak association [54]. In our study, there was no significant difference in cataract prevalence between individuals who were underweight, overweight and obese (Table 2). Our finding that individuals with higher HbA1C are at greater risk of LO aligns with many other studies [40, 41, 52, 55]. Lens damage is attributed to osmotic and oxidative stress and non-enzymatic glycation of lens proteins [56].

The high prevalence of overweight and obesity, and diabetes compared with other countries was also reported in the Phase 5 VBC study [16]. The prevalence of diabetes in our cohort was also higher than other NCD-RisC estimates for India, with little difference between rural and urban populations [16]. In India, the number of people with diabetes is predicted to increase to more than 130 million by 2045 [57], which is likely to further increase the burden of diabetic retinopathy and cataract. The age of onset of diabetes is also generally lower in India than in other populations, which may in part be explained by the ‘thin-fat’ Indian phenotype [58]. India is also undergoing rapid urbanization, with easy access to unhealthy food and reduced levels of physical activity [59]. Considering the inadequate resources for diabetes care and eye care, India faces a huge public eye health problem.

A meta-analysis reported hypertension to be a risk factor for LO, particularly posterior subcapsular opacities [15, 60] but findings were not consistent across studies. Inflammation has been postulated as a likely mechanism. Our estimates (OR 1.73, 95% CI 1.1, 2.7) are comparable to the findings of a meta-analysis of cohort studies (RR 1.08; 95% CI: 1.05–1.12) and case-control or cross-sectional studies (OR 1.28; 95% CI: 1.12–1.45) [60]. Hypertension is also an important risk factor for diabetic retinopathy and could exacerbate the increase in avoidable blindness from cataract and diabetic retinopathy [57].

Despite other studies of sunlight exposure and cataract showing a modest association, including in India [61], sunlight exposure was not significantly associated with LO in our study. This may reflect underestimation of sunlight exposure which was questionnaire based and prone to recall bias.

In the subgroup analysis, vitamin-D deficiency gave a 5-fold higher odds of LO, than those with normal values, which is a stronger association than in other studies [62–65], in South Korean men [65] and in younger women in the USA, for example [64]. Vitamin D deficiency is more frequent in individuals with pigmented skin, lower midday sunlight exposure and those who live at higher latitudes [65, 66]. Photoxidation of lens proteins [67] and altered calcium signalling are implicated in cataractogenesis [68]. LOs in vitamin-D deficiency may be mediated through reduced antioxidant activity [65], and alteration in calcium homeostasis [69]. Lower levels of vitamin D have also been detected in aqueous and vitreous humour in patients with cataract than those with retinal diseases [70]. To our knowledge, this is the first study to show such a strong association between vitamin D deficiency and any cataract in young adults and further studies are warranted.

This is the first observational study of a birth cohort, which provides insights into early ageing manifested by early onset of LOs. The relatively large sample size with rich phenotype and risk factor data adds strength to the study and allows for further follow-up studies of eye conditions. Our study may be limited by selection bias, which is inherent in longitudinal cohort studies where participants can be hard to trace.

Rapid socioeconomic development in India, with changing lifestyles, leading to more obesity, hypertension and diabetes, is likely to increase the burden of cataract blindness in younger adults with alarming public health implications. Modifiable risk factors need to be addressed through eye health promotion, which needs to be integrated into policies and programs for the control of NCDs.

In conclusion, the prevalence of LOs in this birth cohort was higher than in many other studies, but similar to another study in south India. Nuclear cataracts were the commonest form of cataract. A history of bronchial asthma, hypertension and hyperglycaemia were significantly associated with LOs. The strong association with lower serum vitamin D levels needs further investigation in India, as it is a potentially modifiable risk factor.

## Summary

### What was known before

- In Asian Indians, the onset of LOs is earlier than in high income countries, which may reflect earlier onset of ageing.
- Bronchial asthma, hypertension and diabetes are associated with lenticular opacities.

### What this study adds

- A high prevalence of lens opacities in this young population compared with most other studies.
- Further studies are required to explore the role of low serum vitamin D levels which is a potentially modifiable factor.

## Supplementary information

Calculation of Attributable risk between vitamin D deficiency (Yes/No) and lens opacity(Yes/No) (sub-group analysis, n=372)

Comparison of cohort characteristics at 26-32 years (examined in 1998/2002) (n=2218) with whether they were included (n=1075) or excluded (1143) from the lens opacity study at 41-44 years (2013/2014)

## Data Availability

The datasets generated during and/or analysed during the current study are available from the corresponding author on reasonable request.
