# Supplementary material for: Prevalence and risk factors of pre-senile lens opacities in the 1969-73 Vellore Birth Cohort
Source: Eye (Lond). 2025 Jun 13;39(12):2429–37. doi: 10.1038/s41433-025-03836-9 (PMC12325792; doi:10.1038/s41433-025-03836-9)
Supplement: Supplementary file 1 — Calculation of Attributable risk between vitamin D deficiency (Yes/No) and lens opacity(Yes/No) (sub-group analysis, n=372) [file 41433_2025_3836_MOESM1_ESM.docx]

**Supplementary Table 1.**

**Calculation of Attributable risk between vitamin D deficiency (Yes/No) and lens opacity(Yes/No) (sub-group analysis, n=372)**

| Lens opacity (LOCS III) | Vitamin D Deficient (≤20 ng/dL) | |  |
| --- | --- | --- | --- |
|  | Yes | No | Total |
| Yes | 29 | 10 | 39 |
| No | 129 | 207 | 333 |
| Total | 155 | 217 | 372 |

Exposure prevalence (Vitamin D deficiency) = 155/372=41.7% = 0.42

Relative Risk (RR) = 4.1

Attributable risk (AR) = Exposure prevalence in the population (RR-1)/(1+Exposure prevalence in the population (RR-1)

= 0.42x(4.1-1)/1+0.42(4.1-1)

= 1.302/2.302

= 0.56(56%)
