# Supplementary material for: Prevalence and risk factors of pre-senile lens opacities in the 1969-73 Vellore Birth Cohort
Source: Eye (Lond). 2025 Jun 13;39(12):2429–37. doi: 10.1038/s41433-025-03836-9 (PMC12325792; doi:10.1038/s41433-025-03836-9)
Supplement: Supplementary file 2 — Comparison of cohort characteristics at 26-32 years (examined in 1998/2002) (n=2218) with whether they were included (n=1075) or excluded (1143) from the lens opacity study at 41-44 years (2013/2014) [file 41433_2025_3836_MOESM2_ESM.docx]

**Supplementary table 2: Comparison of cohort characteristics at 26-32 years (examined in 1998/2002) (n=2218) with whether they were included (n=1075) or excluded (1143) from the lens opacity study at 41-44 years (2013/2014)**

| **Characteristics** | **Studied in lens opacity study**  **(n=1075)** | **Studied in 1998/2002 study but not in lens opacity study**  **(n=1143)** | **p-value** |
| --- | --- | --- | --- |
| Age(years), Mean (SD) | 28.1 (1.1) | 28.2 (1.3) | 0.086 |
| Sex, n(%)  Male | 577 (53.7) | 584 (51.1) | 0.224 |
| Place of birth, n (%)  Rural  Urban | 573 (53.3)  502 (46.7) | 992 (86.8)  151 (13.2) | <0.001 |
| Education, n (%)  Illiterate/no school  Primary school/literate  Middle school  High school  Above high school | 108 (10.1)  187 (17.4)  261 (24.3)  300 (27.9)  219 (20.4) | 106 (9.3)  197 (17.2)  239 (20.9)  375 (32.8)  226 (19.8) | 0.107 |
| Household Possession score (SES)†  1 (lowest quartile)  2  3  4 (highest quartile) | 252 (23.4)  250 (23.3)  263 (24.5)  310 (28.8) | 324 (28.4)  291 (25.5)  292 (25.6)  236 (20.7) | <0.001 |

SES = socioeconomic status

† Household possession score was obtained using principal component score divided into quartiles
